# Efficacy and Acceptability of *My Care Hub* Mobile App to Support Self-Management in Australians with Type 1 or Type 2 Diabetes

**DOI:** 10.3390/ijerph17072573

**Published:** 2020-04-09

**Authors:** Mary D. Adu, Usman H. Malabu, Aduli E. O. Malau-Aduli, Aaron Drovandi, Bunmi S. Malau-Aduli

**Affiliations:** 1 College of Medicine and Dentistry, James Cook University, Townsville QLD 4811, Australia; mary.adu@my.jcu.edu.au (M.D.A.); usman.malabu@jcu.edu.au (U.H.M.); aaron.drovandi@jcu.edu.au (A.D.); bunmi.malauaduli@jcu.edu.au (B.S.M.-A.); 2 College of Public Health, Medical and Veterinary Sciences, James Cook University, Townsville QLD 4811, Australia

**Keywords:** mobile health, mobile phone applications, diabetes self-management, diabetes education and support, skills, self-efficacy

## Abstract

The aim of this study was to evaluate the preliminary efficacy and user acceptance of *My Care Hub (MCH)* mobile app—developed to provide evidenced-based support and education on diabetes self-management (DSM). Using a mixed-methods design, the efficacy and acceptability of MCH were measured among people with type 1 or type 2 diabetes after three weeks of intervention. The primary outcome measure was level of involvement with DSM, while the mediating factors were skills and self-efficacy for DSM. Telephone interviews were conducted to elucidate information on perceptions of the app’s impact on participants’ DSM and interest in future use. Statistically significant improvements were observed between pre- and post-intervention measures: DSM activities (4.55 ± 1.14 vs. 5.35 ± 0.84; *p* = 0.001); skills (7.10 ± 1.99 vs. 7.90 ± 1.67; *p* = 0.04); and self-efficacy (7.33 ±1.83 vs. 8.07 ± 1.54; *p* = 0.03). Multivariate analysis showed that self-efficacy had the strongest, though not significant influence on DSM. Interview findings revealed that the app reinforced knowledge and provided motivation to participate in DSM activities. The study suggested a positive impact of MCH on DSM and acceptability by patients. To confirm these promising results, further large scale and long-term studies are required.

## 1. Introduction

Diabetes self-management education and support (DSMES) is an ongoing process beyond the formal self-management training, which facilitates the knowledge, skills, and ability necessary for lifestyle behaviours that assist patients to manage their condition [1,2]. This is essential to prevent or reduce the risk of developing complications thus fostering improved short- and long-term health outcomes [3]. Currently, there are complex interplays between barriers and ongoing support for diabetes patients. Barriers include economic, geographical and time constraints for patients, and workforce shortages required to support patients beyond irregular diabetes self-management education classes by health professionals [4,5]. In Australia, patients living in rural and remote areas are more severely impacted by these barriers [6], leading to significant gaps in service delivery, accessibility [7] and lower health outcomes [8]. The Australian Institute of Health and Welfare defines rural as any area outside Australia’s major cities [9], and a significant proportion of Australians with diabetes live in these rural areas [10]. Health system limitations in rural areas highlighted the key role that mobile health (mHealth) interventions, such as applications (apps) play in the provision of ongoing DSMES to patients [11].

Numerous apps aimed at improving self-management activities exist for patients with type 1 or type 2 diabetes. However, many diabetes apps lack explicit description of the development process and design [12], as well as educational components that enhance patients’ knowledge for behavioural change [12,13]. There is also limited consideration of users’ preferences which is necessary to improve the usage of the intervention and behavioural engagement in self-management [14,15]. Furthermore, there has been poor integration of the mediating factors that underpin reported self-management (behavioural) or clinical outcomes in studies while using diabetes app interventions [16,17,18,19]. Factors, including knowledge of DSM skills and self-efficacy (confidence), are important mediators in behavioural change outcomes [20,21]. Thus, integration of these factors into interventions could foster patients’ engagement with DSM. Adequate self-management skills are provided through knowledge about the disease and understanding of the relationship between various self-management behaviours and resulting health outcomes [22]. Self-efficacy, on the other hand, develops patients’ confidence to perform these behaviours and overcome barriers that prevent the achievement of behavioural goals [23].

Previous studies have reported linear positive associations between levels of DSM skills and self-efficacy and participation in specific self-management behaviour, such as diet control, monitoring of blood glucose [24], physical exercise [25,26], foot care [27], as well as overall self-management behaviours [23,28,29,30,31]. This implies that participation in disease self-management behaviours is an end-product of an individual’s management skills and confidence to perform the behaviour. Therefore, a diabetes mHealth intervention aimed at behavioural change should target patients’ improvements in the mediating factors of skills and self-efficacy, which could consequently trigger improved diabetes self-management behaviours.

Researchers have also indicated a positive association between the acceptability of a technology and improved levels of self-management [32,33]. The content and quality of mHealth technology have implications for its acceptability [34], which is an antecedent of users engagement and a key consideration for implementation into practice [35].

Drawing on this background, a new diabetes app intervention called My Care Hub (MCH), was developed to provide evidenced-based support and education to foster self-management behavioural change in Australians with Type 1 or Type 2 diabetes [36]. MCH provide multiple features and functions targeting the mediating factors of skills and self-efficacy in patients to foster improved behavioural change. These features/functions include: (i) an electronic diary and analytics to self-monitor behavioural activities such as blood glucose, physical activities, food intake and weight; and (ii) various educational modules.

### Study Aims

This study reports the preliminary efficacy of MCH. The primary outcome measure is diabetes self-management behaviour pre- and post-intervention. Determinants factors which underpin the process of the primary outcome include changes in diabetes management skills and self-efficacy. In addition, we assessed the acceptability of the app among patients. We posit that the use of MCH in this short-term trial would be acceptable and foster modest improvement in diabetes self-management behaviours due to improvement in skills and self-efficacy.

In the next sections, the methods describing the study design, intervention development, measures of primary outcomes, and mediating variables as well as the results and conclusions drawn from the study will be presented.

## 2. Materials and Methods 

The study procedures were approved by the Human Research Ethics Committee of the James Cook University (reference #H7716). The participants were informed about the study aims and the use of their de-identified data for analysis. Informed consent was implied by submission of an online survey while verbal consent was obtained for all telephone interviews.

### 2.1. Study Design

Details of the study methodology have been fully described in a previous publication [37]. In brief, this pilot study (conducted in August to October 2019) employed a mixed-method sequential explanatory design, where participants accessed the intervention over a three-week period and communicated their perceptions through surveys and interviews. The quantitative phase involved a single-arm repeated measures design entailing the assessment of (1) preliminary efficacy of the intervention through measures of diabetes self-management (DSM) activities, skills, and self-efficacy, where the data were collected online before and after the intervention and (2) the app’s acceptability. The qualitative phase involved telephone interviews with a subsample of participants and it was aimed at gaining greater insight into the role that MCH played in their DSM during the intervention period.

The participants were recruited via an email circulated to people interested in research who were registered with the National Diabetes Service Scheme, Australia. The inclusion criteria were (a) diagnosed with type 1 or type 2 diabetes, (b) aged 18–65 years, (c) live in North Queensland (a rural/regional part of Australia), (d) have a current recommended blood glucose level (BGL) target of 4–10 mmol/L, (e) not pregnant, (f) able to perform activities of daily living, (g) have an android smartphone, and (h) not currently using an app that provides educational support related to DSM. To minimize response bias, a three-staged selection process was used: (i) all invited prospective participants were provided a link to the study information page containing details of the study focus and eligibility; (ii) those who indicated interest and gave consent were directed to the screening questions to confirm that they met all eligibility criteria; (iii) only those who met all of the eligibility criteria were then directed to fill the pre-intervention survey that examined participants’ demographic characteristics and health profile as well as their DSM, skills and self-efficacy levels. A total of 50 participants were enrolled into this study, which is sufficient for a preliminary efficacy study [38].

After filling the pre-study survey, participants were emailed a unique username and password to access the app and its user manual. Participants were provided with technical support to tackle any problems with the app and respond to queries.

### 2.2. Intervention

As outlined in the MCH development protocol [36], the app was specifically designed for those who have type 1 diabetes with recommended blood glucose levels (BGL) of 4–8mmol/L-fasting and <10 mmol/L-2 h postprandial, and fasting levels of 6–8 mmol/L and 2 h post-prandial levels of 6–10 mmol/L for those with type 2 diabetes. Self-efficacy (confidence) construct of the social cognitive theory [39] and the information, motivation constructs of the Information Motivation Behavioural Skills (IMBS) model [40] were the two underlying health behavioral change theories employed in the development of MCH. In the context of this study, the blended concepts of the theories provided mediators for behavioural change. We hypothesised that diabetes self-management behavioural change is mediated by an individual’s self-efficacy which is related to their level of skills to undertake specific tasks required for reaching a desired goal (diabetes self-management).

In relation to the framework described above, “Documentation” and “Analytic” features to monitor BGLs, physical activities and food intake were provided in MCH as techniques to facilitate self-efficacy and consequently improve DSM in patients. Furthermore, the app’s educational modules were developed using the three constructs of IMBS: Information, Motivation and Behavioural Skills. The IMBS model postulates that behavioural change occurs as a result of changes in skills sequel to effect of ‘information’ and ‘motivation’ interventions. Features on the “overview of diabetes management” and “carbs in foods” provide information on diabetes and its self-management. Specifically, actionable ‘information’ on lifestyle modifications (healthy eating and physical activity), monitoring of BGL, complying with medications, good problem-solving skills, healthy coping skills and risk-reduction behaviours (such as smoking cessation, alcohol intake reduction and foot care) for DSM [3] were inputted into the app. In addition, the app contains ‘information’ regarding the approximate equivalent carbohydrate and calorie content of common foods in Australia based on portion sizes of each food.

**Figure 1:**
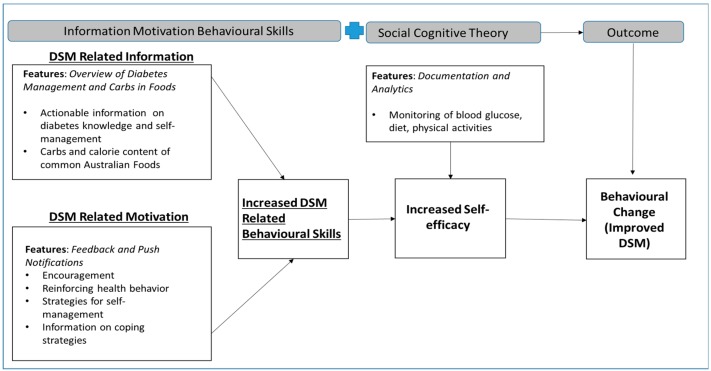
Conceptual framework for My Care Hub based on blended Social Cognitive and Information Motivation Behavioural Skills (IMBS) models.

**Legend:** Interventions on diabetes self-management information and motivation were provided in order to increase participants’ skills for self-management aimed at fostering self-efficacy and consequently improved diabetes self-management behaviours.

In relation to ‘motivation’, this was targeted using the “Feedback” and “Push notification” features in the app. Logged BGL data were automatically evaluated following the Australian Diabetes care guideline’s targeted values: optimally for people with type 1 DM, BGL 4–8 mmol/L before breakfast and <10 mmol/L 2 h after each meal; for people with type 2 DM, BGL 6–8 mmol/L before breakfast and 6–10 mmol/L after each meal. The feedback feature determines if each data item satisfies the guideline requirements or not and then provides feedback in the form of motivational encouragement, advice on lifestyle modifications, or reinforcing health behaviours, as applicable. Lastly, push notifications were provided to strengthen the healthy coping necessary for improved engagement in DSM activities [41]. Notifications provided messages related to diabetes distress, the importance of acknowledging it if experienced by participants, and its’ potential impacts. Participants were then advised to identify realistic goals and focus on them in order to alleviate the distress, which consequently impact their DSM and health outcomes. Examples of actionable goals were provided in order to foster comprehension and engagement. Short, simple text notifications were sent at 12:30 pm once daily during the intervention period. Patients may perceive long and frequent notifications as intrusive and annoying and might limit the opportunity for engagement with the intervention [42]. Although push notifications on apps can provide intervention content to users in a way that can be relatively difficult to ignore [42], we took steps to increase the probability that all participants viewed the messages in order to equalize this intervention dosage. Hence, message sets sent in the first week were reshuffled and resent in the second week. This technique ensured that the participants viewed messages—if a particular message was not opened in the first week on a specific day, it is likely that it will be opened in the second week when sent on a different day. Figure 1 illustrates the conceptual framework for the development and evaluation of the efficacy of the MCH, which was informed by the mediating constructs of social cognitive and IMBS models.

### 2.3. Instrument and Data Collection

Baseline demographic and health characteristics reported by participants included age, gender, employment status, educational level, health care practitioner’s recommended fasting and 2 h post-prandial BGLs, duration since diagnosis and self-perceived health status.

### 2.4. Measures of Primary Outcome and Mediators for Preliminary Efficacy

The primary outcome was frequency of involvement with DSM activities while mediating factors were diabetes management skills and self-efficacy. Improvement in each of the outcomes was defined by a statistically significant increase between the pre- and post- intervention scores. The measuring tool (questionnaire) consisted of two sections, where section one measured the DSM activities using 10 items from the Summary of Diabetes Self-Care Activity (SDSCA) questionnaire [43]. The SDSCA items covered five DSM behavioural domains: BGL monitoring (two items), healthy eating (four items), regular physical activities (two items), and foot care (two items). Participants were asked to recall their activities for the last seven days and state the number of days they performed the behaviours, after which the mean scores across each activity domain was calculated. Section two of the survey collected data on skills and self-efficacy for managing diabetes using the LMC skills, Confidence and Preparedness Index (SCPI) tool [44]. Only 17 items in the SCPI tool that addressed perceived skills (nine items) and self-efficacy (eight items) were relevant and used in this study. Participants were asked how they perceived their ability and confidence to perform diabetes related activities on diet, exercise, taking medications, managing stress, monitoring blood glucose and complications. Items were rated on a 10-point scale where higher values denoted better skills and self-efficacy. The scales have good internal consistency. In the current study, Cronbach alpha for the skills and self-efficacy items were 0.89 and 0.88, respectively.

### 2.5. Measures of Acceptability

Post-intervention, participants also rated their experience with the app while using a set of 18 relevant items adapted from different tools [45,46,47]. On a scale of 1 (strongly disagree) to 5 (strongly agree), the participants rated their agreement with a series of statements about the app’s acceptability (ease of use, intelligibility, satisfaction, perceived value, intention, and behaviour towards recommendation).

### 2.6. Interviews

A single researcher (AD), well experienced in qualitative research conducted semi-structured interviews within three weeks of participants indicating interest. A semi-structured interview guide was developed for the study and elucidated information on participants’ perceptions of the app’s impact on their DSM and interest in future use. The guide was pilot tested by two researchers (MDA and AD), and the first three interviews were used to reflect on the guide, which was found to be appropriate for data collection in its original form. There was no prior relationship between the participants and any of the researchers. Each interview was audio-taped and transcribed. Data saturation was achieved after completing the 14th interview. However, interview sessions with all consenting respondents (17) were completed to allow for rich documentation. Repeat interviews were not required and there was no post-interview debriefing. The conduct and reporting of the interviews followed the consolidated criteria for reporting qualitative research (COREQ) [48]. (please see Appendix A).

### 2.7. Analysis

All quantitative data were analysed using SPSS version 23 [49]. Descriptive statistics was used to present participants’ demographic characteristics. Outcome measures (pre-and post-intervention data) and the acceptability of the intervention were reported using means and standard deviations (SD). The paired sample t-test and Wilcoxon signed-rank test were used to evaluate changes in the outcomes over three weeks for the normally distributed and non-normally distributed variables, respectively. Effect sizes were calculated while using Eta squared values to show the magnitude of changes in outcomes pre- and post-intervention. In addition, multiple regression analysis was used to estimate the contribution of the mediating factors to participants reported overall DSM levels post-intervention. All mediating factors which increased (either significantly or not) from pre- to post-intervention were included in the regression. Two-tailed with *p* < 0.05 were considered statistically significant.

Audiotaped interviews were transcribed verbatim and coding of text fragments based on contents was performed by two researchers (MDA and AD) independently. Consolidation of codes and grouping into themes was achieved through discussion with a third researcher (BMA). Findings are supported with illustrative quotes.

## 3. Results 

### 3.1. Demographics and Health Statistics

Of the 50 participants initially enrolled, 41 (82%) completed the study, including filling in the post-study survey. Participants were predominantly male (61%), aged between 20–64 years (mean, 49.29 years [SD 12.74] and were Caucasians (92.7%). Most of the respondents were residents in rural areas of North Queensland (70.7%), had a technical college education or higher (78%) and were employed (70.7%). Most had type 2 diabetes (71%), rated their health status as ‘good’ or ‘better’ (63.4%), and were diagnosed with diabetes in the previous five years (56.1%). Participants reported their recommended fasting BGL: mean 6.03 ± 1.35; ranged 4–8 mmol/L and 2 h post-prandial: mean 7.53 ± 1.23; ranged 6–10 mmol/L.

### 3.2. Outcomes

Table 1 shows the total mean scores of the DSM domains and mediators: knowledge of diabetes management skills and self-efficacy. At baseline, self-reported adherence to daily dietary recommendations, engaging in physical exercise, and BGL monitoring were generally performed five days a week while foot check was the lowest at about three days a week. The total mean score across all DSM domains was 4.55 ± 1.44. Comparison between pre- and post-intervention scores shows that adherence to diet, monitoring of BGL and overall DSM significantly improved over time (*p* = 0.04, eta squared = 0.1; *p* = 0.04, eta squared = 0.2 and *p* = 0.001, eta squared 0.24, respectively). In relation to skills and self-efficacy, significantly higher scores were observed in both of these factors after the intervention (*p* < 0.05 for both factors with an overall small effect size of 0.11).

#### Relationship between Mediating Factors and Diabetes Self-Management

Positively strong significant correlations were found between skills and self-efficacy (r = 0.835, *p* < 0.001), where those with high level of skills have high self-efficacy. In addition, self-efficacy was weakly correlated with diabetes self-management (r = 0.285, *p* = 0.07).

Multiple linear regression analysis was conducted to examine the variables that predict DSM. After establishing the assumptions of multiple linear regression, analysis identified the simultaneous contributions of skills and self-efficacy on participants’ level of DSM. These variables predicted 8% of the variation of DSM [F (1, 41) = 1.590, *p* = 0.218, R = 0.08]. While both of the factors did not have a significant relationship with DSM, the result shows that self-efficacy has the strongest influence on DSM (β = 0.478). Details are shown in Table 2.

### 3.3. Acceptability

As presented in Table 3, overall mean ratings for all of the items were above 3 on the 5-point scale; suggesting that participants were satisfied with the app’s ease of use and educational content. They noted that the app facilitated improved awareness and stimulated their interest in DSM activities and assented that MCH could serve as a DSM support tool. Participants also expressed interest in the future use of the app if continually available and would recommend it to a friend or family with a similar health condition.

### 3.4. MCH’s Impact on Diabetes Self-Management and Interest in Future Use

An in-depth understanding of the quantitative findings in relation to MCH’s impact on participants’ DSM abilities during the intervention period was elucidated through the interviews. Three major themes emerged from the interviews: “Reinforced knowledge”, “motivation for self-management”, and “continuity”.

#### 3.4.1. Reinforced Knowledge 

Participants perceived that the educational messages in MCH reinforced their knowledge about diabetes and self-management of the condition.

*“I did have some knowledge as I have been to a dietician. But with the app. It is always good to have that little message to reinforce you each day to watch out for things that you shouldn’t have too much of”*.

[P014, T2D]

*“It sort of helped me and remind me of watching the diet. High blood glucose level is a reflection of what you consume. It reminded me in that regard to be careful of what I eat”*.

[P009, T2D]

The messages also prompted reflection on how best to handle events/situations.

*“It clarifies the information I already know because this is a sort of disease that you can’t see. It is eating away in the background there and the app lets you look at it from a different way other than just pricking your finger three or four times a day and prick again and it is still high. With the app, I kind of try to keep it under control”*.

[P011, T2D]

A few participants reported that apart from the app reinforcing their knowledge, they also gained new information related to the effect of diabetes distress on blood glucose.

*“Some of it was new information. It was quite interesting to know how stress affects diabetes and your sugar. I have a bit of stress every now and then. That information is something I had never thought about”*.

[P016, T2D]

When asked about the advantage of promoting this intervention to a larger population, participants reported that the app would particularly improve the knowledge of people newly diagnosed with diabetes as a result of its educational information component:

*“Especially people who are new to diabetes could get a lot of information from it (MCH). It would help them a lot to sort out what they are doing and what is going on”*.

[P008, T2D]

*“People who are new to diabetes, like after attending a couple of courses, it could help them to understand a lot more”*.

[P10, T2D]

#### 3.4.2. Motivation for Self-Management

Participants described that the app provided motivation to care for themselves and encouraged participation in different aspects of their self-management.

*“Yes, it increased my motivation. I do my blood test and I weigh myself regularly and I was going out to a do a reasonable amount of exercise”*.

[P005, T2D]

Some participants mentioned that MCH gave them some degree of control on managing their condition.

*“For once it was about doing something for me, giving information to me and giving me I would say a degree of control…what I stick in my mouth”*.

[P005, T2D]

Several features in MCH supported different self-management activities and were perceived to improve easy accessibility to necessary support:

*“I think just having everything there at your fingertips, the BGL levels, the exercise, your food, your diet, your carbs counting sort of thing. It was all there for you. You know the flexibility of it”*.

[P009, T2D]

#### 3.4.3. Continuity 

Participants also expressed strong interest in future use and recommendation of the app to other users. Participants reiterated their intention to continue using the app if accessible:

*“If you are serious about looking after yourself and stay within your target blood glucose range, I would definitely say yes to the app”*.

[P007, T2D]

In addition, a participant narrated that his doctor was positive about the app’s content and willing to recommend it to his other patients:

*“I told my doctor I was doing a study and he had a look (at the app) and said yeah, that looks good. He asked what the green things were and I said, the green ones are what you should be eating and the other ones are high in carb. He thought that was OK and wanted to know what it is called because if he had other patients, he said he could direct them to downloading that app”*.

[P13, T2D]

## 4. Discussion

This pilot study investigated the preliminary efficacy and acceptability of the MCH app which was designed to improve participation in DSM in people with type 1 and type 2 diabetes.

### 4.1. Preliminary Efficacy

In this study, patients reported improved levels of participation in all domains of measured DSM activities and this may be due to increased motivation to engage in self-management activities through the use of MCH as reported in the interviews. Few short-term research studies have reported on the preliminary efficacy of mobile phone apps either in relation to overall DSM activities or for single self-management activity change (e.g., dietary or physical activity only). Agarwal et al. [50] and Faridi et al. [51] tested the effect of a mobile technology on overall DSM, while others have monitored diet [52] or physical activity [52] as part of program evaluation for diabetes support. Preliminary efficacy results of these apps vary from none [50] to moderate [51,52] among participants in the intervention settings. Therefore, the significant improvements in DSM observed in our study are unique and they could be termed to have clinical significance when viewed in the context of impact on diabetes management.

In reality, continuing health-care provider support for DSM is not always available. Ongoing DSMES for improved self-management is needed to reduce or prevent the risk of developing complications and other poor health outcomes [2,3], which are particularly prevalent among Australian rural populations [8,53,54]. The provision of a potentially highly effective mobile health app such as MCH for improving DSM could be an important supportive measure among this patient population. The MCH intervention provides educational features, documentation features, Analytics and feedback i.e., guidance based on information entered by users. The use of multi-component behavioural change strategies and mHealth features as described above have the greatest potential impact on behavioural change in self-management [42,55,56].

In recent years, several mHealth applications have been developed in order to support self-management in people with diabetes, with these interventions being deemed feasible and acceptable, though evidence of improved self-management is either unclear or weak [57,58]. This may be due to a lack of proper consideration of the mediating factors that are necessary to produce improved DSM. Adequate skills and self-efficacy are major pivoting mechanisms for behavioural change in diabetes management [20,21,23]. Therefore, the consequent impact of these factors to produce improved DSM is expected and confirmed our hypothesis. Skills is the understanding and ideas that patient possesses about a subject (*diabetes and its management*), potentially with the ability to use it for a specific purpose (self-management) [59], and it fosters self-efficacy—the confidence a patient has in his/her self to achieve the purpose [60]. Self-efficacy is a prerequisite for informed health decision making [61,62] and greatly influences the probability for behavior initiation, level of applied effort and how long behaviour will be sustained [60]. Therefore, the results of this study is a further proof-of-concept, supportive of previous literature on the value of improved self-efficacy to promote behavioural change [63,64,65]. Nevertheless, the non-significant predictive power of self-efficacy on DSM which we found in this study might be an indicator that self-efficacy is not strong enough to make a large effect in a short time frame. As such, it is likely that the strong causal relationship will require more prospective investigations.

Participants in this study emphasised that the intervention motivated them to engage in their DSM as well as reinforced their knowledge of diabetes. MCH educational content serving as just-in-time resource to increase motivation and prompts to action in self-management have also been described in other studies [66,67]. This result suggests that the app could be a feasible means of augmenting self-management education and support. Furthermore, the app was perceived to be a particularly useful tool for people who are newly diagnosed with diabetes to remind them of many issues discussed during face-to-face diabetes education session with their health providers on the importance of self-management and adherence to it for improved health outcomes.

### 4.2. Acceptability

The acceptability of a mHealth technology is an indication of its value and the importance for wider implementation into the healthcare system [35]. The result of this study indicates good level of acceptability of MCH, as most participants endorsed the app components as useful and supportive of their DSM. Other studies assessing the acceptability of mobile apps for diabetes self-management were similarly positive [68,69]. This result might have also fostered higher levels of DSM reported in the post-intervention, because studies have demonstrated a positive association between higher levels of acceptability of a mHealth and self-management [33,70]. Likewise, perceived ease of use and satisfaction with health apps positively affect continued intention of use [71]. These were reflected in our study, as participants expressed overall satisfaction with the simplicity of MCH with intentions of continued use and recommendation to others. Nevertheless, acceptability has been described as only ‘one piece of a puzzle’, because even with high acceptance levels, uptake and upscale of the intervention may diverge. Hence, the recommendation that healthcare providers who perceive strong benefits of mHealth technology should endeavour to encourage patients’ adherence to it [72].

### 4.3. Strength and Limitations

The study utilised mixed-methods research design which allowed for detailed exploration of participants’ experiences and perspectives about the app. In addition, the study provided an explanation of the preliminary efficacy of MCH app on diabetes self-management in relation to its mediating factors as targeted in the intervention. Such report is often lacking in many preliminary efficacy studies of mHealth technologies. The use of theory-driven and evidenced based intervention support strategies is also a notable strength of this study.

There are some limitations to the current research. A longer follow-up period would have provided clearer insights into the sustenance of the reported behaviour changes, however the short-term intervention period in this study is comparable to that of other studies with 2–3 week intervention period [73,74]. Furthermore, our study population were patients registered with NDSS and interested in research, potentially implying many participants were already on top of their self-management, as reflected in the high level of DSM at baseline. This reduces to an extent, the generalisability of the study findings to other populations. Additionally, the tools adapted for measuring acceptability of the intervention were not used in their entirety as items not relevant to the current study were removed. Using only few items from a validated tool might compromise its uniformity. Nevertheless, the selected set of items in each validated tool demonstrated good internal consistencies with Cronbach alpha from 0.70 to 0.91. We noted that our sample size like many similarly published pilot trials was modest. In addition, measured outcomes were self-reported and thus may be subjected to social desirability and recall bias. Lastly, the lack of control group may limit the conclusions that can be made regarding the beneficial impact of the app. Nevertheless, preliminary work such as this is a useful and necessary precursor to more rigorous examination of the intervention in a large-scale trial with longer-term follow up.

### 4.4. Future Directions

#### 4.4.1. Automatic Push Notifications

The education component in MCH specifically delivered through the push notification feature requires human coaching where by a diabetes educator provides daily education through this platform during the intervention period. Intervention that relies on human input requires substantial human resources, which if lacking, may limit the scalability of the intervention. Therefore, further improvement of MCH requires automation of the push notification educational components free of human involvement as much as possible. This will lower the cost of operation and improve scalability of the intervention.

#### 4.4.2. Long-Term Trial

The promising result of this pilot MCH app project which shows preliminary efficacy, acceptability (as reported in this study) as well as good level of retention and engagement with the intervention [37] will require further confirmation using long term controlled trials in the future. An adaptive randomized controlled trial design [75] may be best suited due to the rapidly evolving nature of mHealth. The design will enhance dynamic adaptation of the app to the advancing field of information technology thus facilitating better understanding of the unique impact of each of the app features, thereby fostering improvement and long-term utility of the MCH intervention in the support and management of diabetes.

## 5. Conclusions

The use of mobile phone application intervention among underserved population represents a novel approach to augmenting self-management education and support. We propose an innovative app–MCH, as a self-management tool for Australians with type 1 or type 2 diabetes. The results of this pilot trial suggest that MCH app can be an acceptable and potentially effective intervention that can be replicated in other contexts to improve diabetes self-management. Future work should employ larger and long-term trials to further establish the efficacy of the app and the impact on glycaemic control and other health outcomes.

## Figures and Tables

**Table 1:** Observed mean and standard deviations for the outcome measures.

Outcome	Baseline, Mean (SD)	Post-Intervention, Mean (SD)	*p*	Effect Size
**Diabetes self-management**				
Diet	5.13 (1.10)	5.54 (0.90)	0.04 *	0.10
Physical activity	4.48 (2.16)	5.35 (2.27)	0.09	0.07
Monitoring of BGL ^a^	5.16 (2.81)	6.80 (1.95)	0.04 *	0.20
Foot check	2.87 (1.86)	3.51 (1.79)	0.18	0.05
*Overall*	4.55 (1.14)	5.35 (0.84)	0.001 *	0.24
**Skills and Self-efficacy**				
Skills	7.10 (1.99)	7.90 (1.67)	0.04 *	0.23
Self-efficacy	7.33 (1.82)	8.07 (1.54)	0.03 *	0.25
*Overall*	7.27 (1.83)	8.00 (1.55)	0.04 *	0.11

* *p* < 0.05; ^a^ BGL: Blood glucose levels.

**Table 2:** Influence of mediating variables on diabetes self-management.

Determinant Variables	*B*	*SE*	*Beta*	*t*	*p*
Diabetes management skills	−0.15	0.144	0.298	0.138	0.306
Self-Efficacy	0.26	0.157	0.478	1.664	0.105
**Constant (α) = 4.428**					
R^2^ = 0.079; Adjusted R^2^ = 0.29					

**Table 3:** Participant acceptability ratings (N = 41).

Survey Item	Mean	SD
**Ease of use/intelligibility/satisfaction**		
I feel confident using the app	4.2	0.68
I am satisfied with how easy it is to use the app	3.9	0.83
I felt comfortable using the app	4.02	0.76
I found the educational tips embedded in the app easy to understand	4.07	0.65
I found the immediate feedback provided after my BG log easy to understand	4.15	0.73
The messages displayed through push notification were easy to understand	4.17	0.59
Overall, I am satisfied with the app	3.68	1.04
Total	4.02	0.75
**Value**		
The daily messages (push notifications) increased my awareness of the importance of engaging in my self-care activities	3.59	1.14
The app features could stimulate my interest to continually participate in my self-care and record the activities	3.56	1.16
The app support my self-care such as tracking of BG, provide an idea of the carb content of my food	3.8	1.03
The daily messages (push notifications) motivates me more to pay attention to managing my diabetes	3.46	1.14
I found the immediate feedback received after logging my BG helpful for my self-management	3.61	1.16
The notifications motivates me to do my self-care activities (e.g., exercise, healthy eating, BG monitoring)	3.41	1.16
My Care Hub app could serve as a self-management support tool for people with diabetes	4.05	0.87
Total	3.64	1.09
**Intention for use and recommendation**		
If I have continual access to the app, I will use it frequently	3.46	1.23
I think I would like to use the app more frequently	3.49	1.23
I could recommend the app to family and friends who have my type of diabetes	3.66	1.15
If I were to proceed with the program, I want to receive fewer push notification messages	3.02	1.01
Total	3.40	1.16

## Abbreviations

BGL	Blood glucose levels
DSM	Diabetes self-management
DSMES	Diabetes self-management education and support
MCH	My Care Hub
NDSS	National Diabetes Service Scheme
RCT	Randomised controlled trial

## Appendix A

Consolidated criteria for reporting qualitative research checklist. Available in the Table A1.

**Table A1:** Consolidated criteria for reporting qualitative studies (COREQ): 32-item checklist.

No. Item	Guide Questions/Description	Where in Manuscript
**Domain 1: Research team and reflexivity**		
*Personal Characteristics*		
1. Interviewer/facilitator	Which author/s conducted the interview or focus group?	AD
2. Credentials	What were the researcher’s credentials? e.g., PhD, MD	MDA: *BSc, Msc, Grad Cert Diab Edu*; UHM: MBBS, *Msc, MD*; AEOMA: *BSc, Msc, PhD;* AD: Bsc, *PhD* BSMA: *BSc, Msc, Grad Cert ULT, Grad Cert Mgt, PhD*
3. Occupation	What was their occupation at the time of the study?	Please find at the end of this list
4. Gender	Was the researcher male or female?	MDA: Female UHM: Male AEOMA: Male AD: Male BSMA: Female
5. Experience and training	What experience or training did the researcher have?	All authors were experienced researchers in qualitative studies and have taken part and published peer reviewed articles in mHealth for diabetes management.
*Relationship with participants*		
6. Relationship established	Was a relationship established prior to study commencement?	Methods
7. Participant knowledge of the interviewer	What did the participants know about the researcher? E.g., personal goals, reasons for doing the research	Methods
8. Interviewer characteristics	What characteristics were reported about the interviewer/facilitator? E.g., Bias, assumptions, reasons and interests in the research topic	The motivation and background of the study were made clear to the participants before the start of the interview. Participants had no prior relationship with the interviewer.
**Domain 2: study design**		
*Theoretical framework*		
9. Methodological orientation and Theory	What methodological orientation was stated to underpin the study? e.g., grounded theory, discourse analysis, ethnography, phenomenology, content analysis	Methods
*Participant selection*		
10. Sampling	How were participants selected? e.g., purposive, convenience, consecutive, snowball	Methods
11. Method of approach	How were participants approached? e.g., face-to-face, telephone, mail, email	Methods
12. Sample size	How many participants were in the study?	Results
13. Non-participation	How many people refused to participate or dropped out? Reasons?	Results
*Setting*		
14. Setting of data collection	Where was the data collected? e.g., home, clinic, workplace	Methods
15. Presence of non-participants	Was anyone else present besides the participants and researchers?	Methods
16. Description of sample	What are the important characteristics of the sample? e.g., demographic data, date	Results
*Data collection*		
17. Interview guide	Were questions, prompts, guides provided by the authors? Was it pilot tested?	Methods
18. Repeat interviews	Were repeat interviews carried out? If yes, how many?	Methods
19. Audio/visual recording	Did the research use audio or visual recording to collect the data?	Methods
20. Field notes	Were field notes made during and/or after the interview or focus group?	None
21. Duration	What was the duration of the inter views or focus group?	Methods
22. Data saturation	Was data saturation discussed?	Methods
23. Transcripts returned	Were transcripts returned to participants for comment and/or correction?	Methods
**Domain 3: analysis and findings**		
*Data analysis*		
24. Number of data coders	How many data coders coded the data?	Methods
25. Description of the coding tree	Did authors provide a description of the coding tree?	Methods
26. Derivation of themes	Were themes identified in advance or derived from the data?	Methods
27. Software	What software, if applicable, was used to manage the data?	Methods
28. Participant checking	Did participants provide feedback on the findings?	Methods
*Reporting*		
29. Quotations presented	Were participant quotations presented to illustrate the themes/findings? Was each quotation identified? e.g., participant number	Results
30. Data and findings consistent	Was there consistency between the data presented and the findings?	Yes, Results
31. Clarity of major themes	Were major themes clearly presented in the findings?	Yes, Results
32. Clarity of minor themes	Is there a description of diverse cases or discussion of minor themes?	Yes, Results

**Occupation of interviewer and researchers at the time of the study:**

- Mary D. Adu: PhD Candidate, College of Medicine and Dentistry, James Cook University. Australia.
- Usman H. Malabu: Consultant Endocrinologist and Professor of Medicine, Townsville Hospital and Health Services/College of Medicine and Dentistry, James Cook University. Australia
- Aduli E. O. Malau-Aduli: Associate Professor, College of Public Health, Medical and Veterinary Sciences, James Cook University.
- Aaron Drovandi: Lecturer, College of Medicine and Dentistry, James Cook University. Australia
- Bunmi S. Malau-Aduli: Associate Professor, College of Medicine and Dentistry, James Cook University. Australia.
